# Antidermatophytic Action of Resorcinol Derivatives: Ultrastructural Evidence of the Activity of Phenylethyl Resorcinol against *Microsporum gypseum*

**DOI:** 10.3390/molecules21101306

**Published:** 2016-09-30

**Authors:** Carlo Romagnoli, Anna Baldisserotto, Chiara B. Vicentini, Donatella Mares, Elisa Andreotti, Silvia Vertuani, Stefano Manfredini

**Affiliations:** 1 Department of Life Sciences, University of Modena and Reggio Emilia, Viale Caduti in Guerra 127, 41121 Modena, Italy; carlo.romagnoli@unimore.it (C.R.); elisa.andreotti@unimore.it (E.A.); 2 Department of Life Sciences and Biotechnology, Master Course in Cosmetic Science and Technology, University of Ferrara, Via Fossato di Mortara 17-19, 44121 Ferrara, Italy; bldnna@unife.it (A.B.); chiara.vicentini@unife.it (C.B.V.); donatella.mares@unife.it (D.M.); silvia.vertuani@unife.it (S.V.)

**Keywords:** resorcinol derivatives, dermatophytes, *Microsporum gypseum*, antifungal activity, TEM, SEM

## Abstract

In this work, we evaluated the antidermatophytic activities of three resorcinol derivatives that have a history of use in dermo-cosmetic applications to discover molecules with multiple dermatological activities (i.e., multi-target drugs), thereby reducing the cost and time necessary for new drug development. The antidermatophytic activities of the three skin lighteners were evaluated relative to the known antifungal drug fluconazole on nine dermatophytes responsible for the most common dermatomycoses: *Microsporum gypseum*, *Microsporum canis*, *Trichophyton violaceum*, *Arthroderma cajetani*, *Trichophyton mentagrophytes*, *Epidermophyton floccosum*, *Nannizzia gypsea*, *Trichophyton rubrum* and *Trichophyton tonsurans*. Among the three tested resorcinols, only two showed promising properties, with the ability to inhibit the growth of all tested dermatophytes; additionally, the IC_50_ values of these two resorcinols against the nine dermatophytes confirmed their good antifungal activity, particularly for phenylethyl resorcinol against *M. gypseum*. Ultrastructural alterations exhibited by the fungus were observed using scanning electron microscopy and transmission electron microscopy and reflected a dose-dependent response to treatment with the activation of defence and self-preservation strategies.

## 1. Introduction

We recently initiated a systematic study [1] that utilizes a multi-target drug discovery approach to discover new molecules with antifungal activities among drugs already used for other dermatological applications. The objective of this study is to discover novel antifungals with low toxicity, as proven by their long history of dermatological use. In particular, our attention was drawn to resorcinols, which are naturally occurring phenolic compounds that are mainly synthesized by plants; these molecules are widely used in the dermatology and cosmetic fields as skin lighteners and have a very good safety profile.

Resorcinol has a long history of therapeutic use for its keratolytic properties [2] and has been included, usually with sulphur, in topical preparations for the treatment of acne and seborrhoeic skin conditions, although other treatments are generally preferred [2].

Resorcinol is one of the components in the well-known Castellani’s solution, which was developed in 1905 by Aldo Castellani, an Italian doctor [3]. The traditional Castellani’s solution contains boric acid, phenol, fuchsine, resorcinol, acetone, alcohol and water. Its antifungal activity is attributed to fuchsine, and its antibacterial activity is ascribed to ethanol. However, the role of resorcinol is also intriguing. In fact, in the last few years, increasing data have indicated that the compound has various biological activities, including antimicrobial, antiparasitic and cytotoxic activities [4,5], such as antioxidant (anti-inflammatory) and antigenotoxic activities [6].

**Figure 1:**
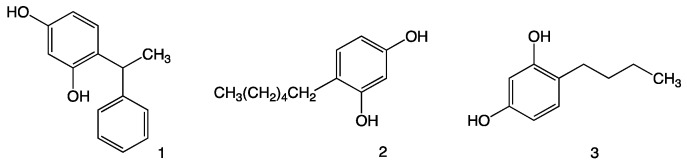
Chemical structures of (±)-phenylethylresorcinol (**1**), 4-hexylresorcinol (**2**) and 4-butyl-resorcinol (**3**).

We selected three compounds in this class—(±)phenylethylresorcinol (**1**), 4-hexylresorcinol (**2**) and 4-butylresorcinol (**3**) (Figure 1)—based on their widespread use in dermatology and investigated their effects against nine dermatophytes. To the best of our knowledge, these compounds are not currently used as anti-dermatophytes, and rather they are currently employed as skin-lightening agents because they inhibit tyrosinase [7,8].

The results of a preliminary screening were encouraging: plate tests and germination tests showed good activity, with high inhibition rates against many fungi. These favourable data emphasized the need for a deeper investigation of the antifungal activities of these substances, including determination of their IC_50_ values, which reflect the effectiveness of a compound. These measurements were made for the two compounds that gave the best preliminary results.

Subsequently, the mechanism of action of the most active molecule, phenylethylresorcinol, against *Microsporum gypseum* (Bodin) Guiart & Grigorakis, was investigated by electron microscopy. This fungus was chosen based on its IC_50_ values. The IC_50_ values of the selected resorcinols against *M. gypseum* were the lowest among all the fungi tested, indicating that this organism is highly sensitive to the treatment.

*M. gypseum* is a geophilic fungus. It is a component of the complex soil mycoflora and is frequently found in soils rich in organic matter. It is distributed worldwide and infects humans, particularly children and rural workers during warm humid weather, producing circinate herpes and tinea capitis [9,10].

Lesions caused by *M. gypseum* frequently induce strong inflammatory reactions that mimic dermatitis. This dermatitis is often treated with steroids to reduce inflammation, resulting in an atypical appearance as tinea. It must also be noted that the risks associated with modern lifestyle activities, e.g., cosmetic tattooing and pet keeping, have increased interest in the discovery of novel multi-target drugs against *M. gypseum* [11].

The available literature on *M. gypseum* is fairly sparse. In the current study, we investigated the fungus using scanning electron microscopy and transmission electron microscopy (SEM and TEM, respectively).

## 2. Results

### 2.1. Antifungal Activity

Table 1 shows the growth inhibition induced by the three tested resorcinol derivatives against nine dermatophytes. Notably, 4-butylresorcinol showed only low inhibition against three dermatophytes (*M. gypseum, T. mentagrophytes* and *E. floccosum*), even at the two highest doses.

Both 4-hexylresorcinol and phenylethylresorcinol generally showed very good antifungal activity against the nine dermatophytes at all concentrations. Both substances exhibited nearly 50% inhibition of almost all fungi, even at a concentration of 50 μg/mL; however, against *M. gypseum*, both exceeded 50% inhibition at a concentration of 20 μg/mL. Only phenylethylresorcinol achieved 100% inhibition at the highest concentration against three dermatophytes (*T. violaceum, A. cajetani* and *T. mentagrophytes*), whereas against *T. tonsurans*, it caused 100% inhibition at concentrations as low as 100 μg/mL.

Based on these results, we decided to more thoroughly investigate phenylethylresorcinol. This compound may be of potential use in antifungal therapy because of its increased activity against the nine dermatophytes tested, shown in Table 2, in comparison with that of the well-known antifungal agent fluconazole. The results clearly show that fluconazole was less active than phenylethyl-resorcinol.

In addition, the two resorcinol derivatives identified as the most active in the previous screening were also evaluated for their potency, as reported in Table 3.

The IC_50_ values were low for almost all tested fungi. *T. violaceum* was the least inhibited with phenylethyl resorcinol, whereas the most inhibited fungi was *M. gypseum* (11.42 μg/mL).

### 2.2. Inhibition of Spore Germination by Resazurin Assay

**Figure 2:**
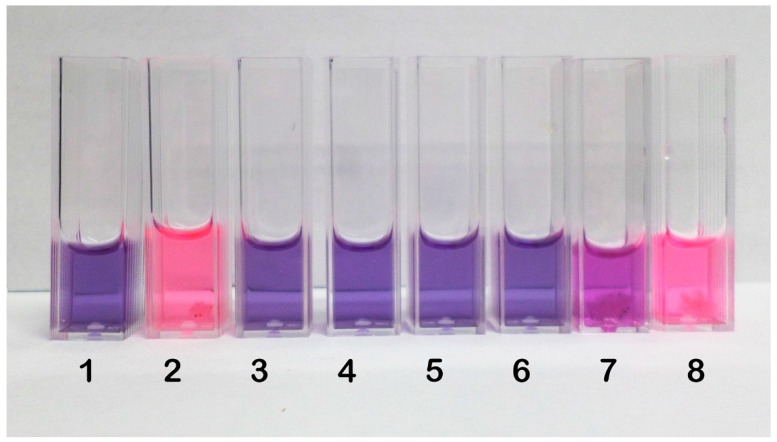
Colours of the cuvettes after the resazurin assay. Legend: **1**—negative control; **2**—positive control; **3**—phenylethylresorcinol (200 μg/mL); **4**—phenylethylresorcinol (100 μg/mL); **5**—4-hexylresorcinol (200 μg/mL); **6**—4-hexylresorcinol (200 μg/mL); **7**—4-butyl-resorcinol (200 μg/mL); **8**—4-butylresorcinol (100 μg/mL).

As *M. gypseum* was the most sensitive fungus to the three substances, it was tested for spore germination inhibition. As shown in Table 4, the results of the test confirmed the excellent inhibitory capacities of phenylethylresorcinol and 4-hexylresorcinol, as evidenced by the blue staining of the cuvettes, which indicated the absence of viable spores (Figure 2).

In contrast, 4-butylresorcinol inhibited spores germination at different stages, as indicated by the pinkish colour of the test cuvettes. Given these results, we chose the most active compound (phenylethylresorcinol) and the most sensitive fungus (*M. gypseum*) for electron microscopic evaluations.

### 2.3. SEM Observations

**Figure 3:**
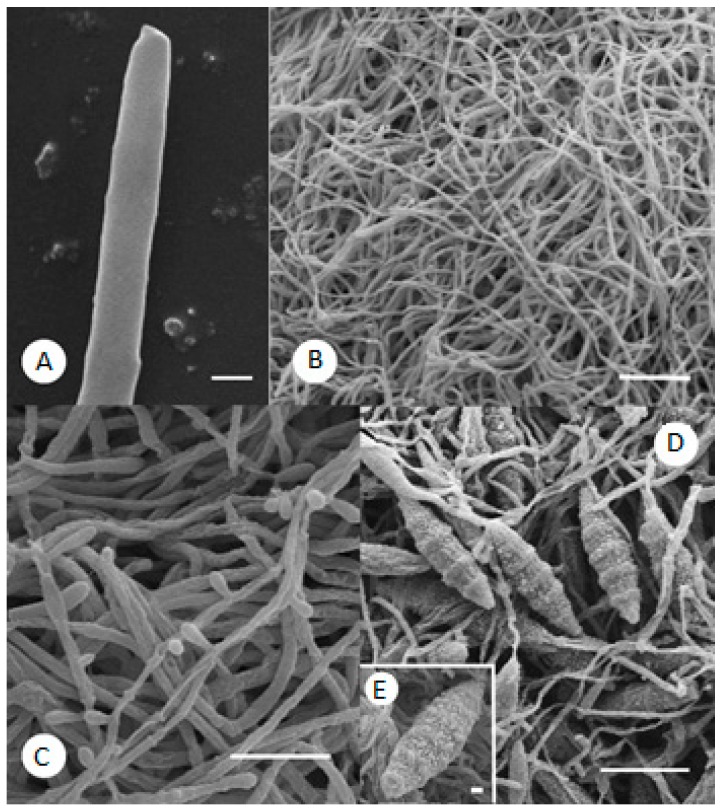
(**A**) Control *M. gypseum*: detail of straight apex with smooth wall. SEM. Scale bar: 1 µm; (**B**) Same sample: mycelium without visible microconidia. SEM. Scale bar: 20 μm; (**C**) *M. gypseum* treated with 20 μg/mL phenylethylresorcinol: microconidia are visible. SEM. Scale bar: 10 μm; (**D**) *M. gypseum* treated with 100 μg/mL phenylethylresorcinol: numerous macroconidia in the mycelium. SEM. Scale bar: 20 μm; (**E**) Same sample: typical cigar-shaped macroconidium with knobs on the surface. SEM. Scale bar: 2 μm.

The control mycelium of *M. gypseum*, (Figure 3A) showed a typical morphology, exhibiting lengthened hyphae of constant diameter with a rounded or lightly tapering apex and smooth external surface. No microconidia or macroconidia were visible (Figure 3B) in the mycelium, as is expected for a young culture of 24 h.

In samples treated with 20 μg/mL phenylethylresorcinol, the appearance of the mycelium was similar to that of the controls, except for the numerous microconidia (Figure 3C). In contrast, at 100 μg/mL, the microconidia were no longer visible, although the mycelium contained abundant macroconidia (Figure 3D) showing the typical “cigar shape” with characteristic knobs on the outer surface (Figure 3E).

These alterations on *M. gypseum* treated with phenylethylresorcinol are very interesting because they potentially reflect the development of defence strategies. As reported in the literature on other dermatophytes [12], an increase in the number of reproductive structures represents a defensive response by the microorganism. The production of microconidia or macroconidia is a form of asexual reproduction that occurs in an attempt to overcome nutrient starvation [13].

**Figure 4:**
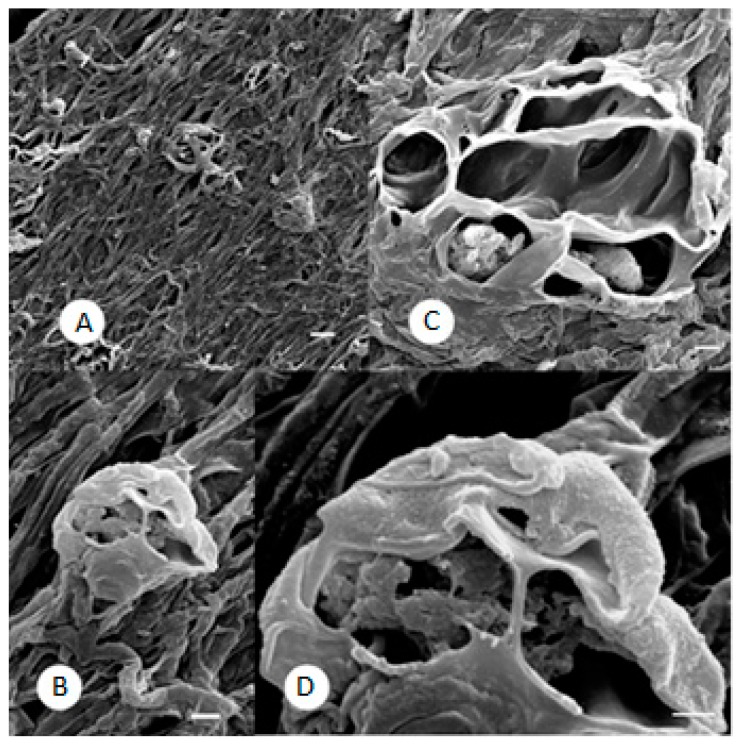
*M. gypseum* treated with 200 μg/mL phenylethylresorcinol. (**A**) Compact mycelium with hyphae fused together and some stretch marks. SEM. Scale bar: 10 μm; (**B**) Strange formations in the compact mycelium with the appearance of burst balloons. SEM. Scale bar: 2 μm; (**C**) Cave formations, probably derived from hyphae fusion, with spongy material visible inside. SEM. Scale bar: 2 μm; (**D**) High magnification of one of the exploded structures, showing the typical roughness of a macroconidia on the external surface. SEM. Scale bar: 1 μm.

At the lower doses tested, the substances only exhibited fungistatic activities, concordant with the literature on the antifungal activities of resorcinol derivatives against phytopathogens [6]. Conversely, at the highest concentration tested (200 μg/mL), the mycelium presented notable morphological abnormalities. This concentration probably overwhelms the defensive capabilities of the fungus, inducing the rupture or bursting of its defensive structures. At low magnification, the mycelium appears highly compact (Figure 4A), with hyphae fused together to form a singular woven structure that is occasionally interrupted by areas with the appearance of “stretch marks”. Neither micro- nor macroconidia are visible, but in addition to crushed and very suppressed hyphae, strange formations with the appearance of burst balloons can be observed (Figure 4B).

At the highest resolution (Figure 4C), in the layers underlying the cave formations, a type of trabecula can be observed that seems to be derived from the fusion of hyphae. The presence of material with a “spongy” consistency in some of these abnormal bodies exhibiting several openings is noteworthy.

In Figure 4D one of these bodies has on its outer surface the typical roughness or blebbing of macroconidia. The highest concentration of phenylethylresorcinol (200 μg/mL) may have exerted a fungicidal action by blocking fungal defence strategies.

### 2.4. TEM Observations

**Figure 5:**
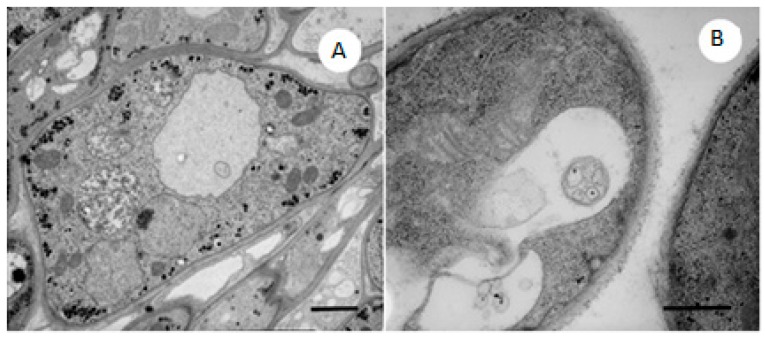
(**A**) *M. gypseum* control with normal mitochondria, nuclei and ribosomes. Note the glycogen rosettes near the plasmalemma. TEM. Scale bar: 1 μm; (**B**) *M. gypseum* treated with 20 μg/mL phenylethylresorcinol. Glycogen, which is no longer arranged in rosettes, is scattered throughout the cytoplasm, and there is an increased number of large vacuoles. TEM. Scale bar: 0.5 μm.

Figure 5A shows a typical structure of the *M. gypseum* control, with glycogen rosettes close to the plasmalemma. The cell wall is normally structured with two typical layers visible: the outer, non-electron-transparent, mannoproteic layer and the inner, translucent layer composed primarily of glucan and chitin (Figure 5A,B).

In the samples treated with the lowest concentration of phenylethylresorcinol (20 μg/mL), the most evident feature is the increased number of large vacuoles. Glycogen, which is no longer visible as rosettes, is scattered throughout the cytoplasm (Figure 5B), and some normally structured mitochondria are visible.

**Figure 6:**
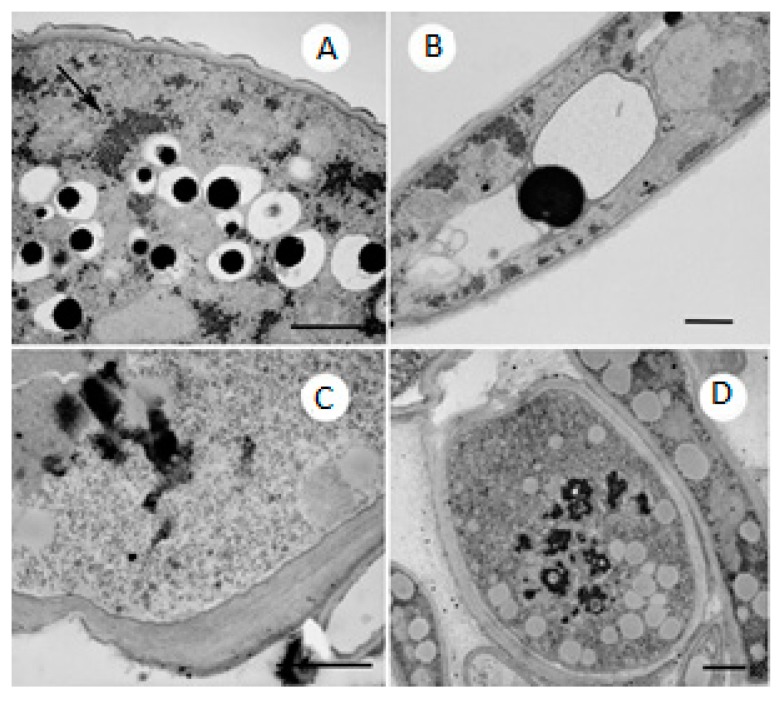
(**A**) *M. gypseum* treated with 100 μg/mL phenylethylresorcinol. Numerous small vacuoles containing non-electron-transparent material. Note the accumulation of glycogen near the vacuoles. TEM. Scale bar: 1 μm; (**B**) Same sample. Non-electron-transparent body in the cytoplasm surrounded by two vacuoles. TEM. Scale bar: 1 μm; (**C**) High magnification of the cell wall of *M. gypseum* treated with 200 μg/mL phenylethylresorcinol. The large increase in the thickness of the cell wall, which contains several layers, is evident. TEM. Scale bar: 1 μm; (**D**) Sample treated with the same. Numerous small vacuoles containing dark, irregularly shaped glycogen bodies. TEM. Scale bar: 1 μm.

At 100 μg/mL, many smaller vacuoles are present and contain non-electron-dense materials. Glycogen is distributed in the cytoplasm, accumulating near the vacuoles (Figure 6A). The TEM images also show ultrastructural changes reflecting the defensive efforts of the fungus. Notably, the number of vacuoles increased, resulting from the activation of autophagic phenomena in filamentous fungi. Autophagy is primarily a pro-survival mechanism in which normal cellular components are absorbed into vacuoles and digested as a major response to nutrient deprivation and stress. However, it can also be induced by antifungal compounds [13,14]. Indeed, the incorporation of glycogen into vacuoles, which was seen at the highest concentration, is a clear autophagic phenomenon that protects against injury [15].

Figure 6B shows the possible formation of non-electron-transparent circular bodies in the cytoplasm that are surrounded by vacuoles and subsequently appear to be segregated, as shown in the previous figure (Figure 6C). Their incorporation into the vacuoles may have the same autophagic purpose. After treatment with phenylethylresorcinol at 200 μg/mL, the most evident feature is the large increase in the thickness of the cell wall, which appears to be at least twice as thick as that of the control. This phenomenon can be seen in Figure 6C, where several layers are evident.

**Figure 7:**
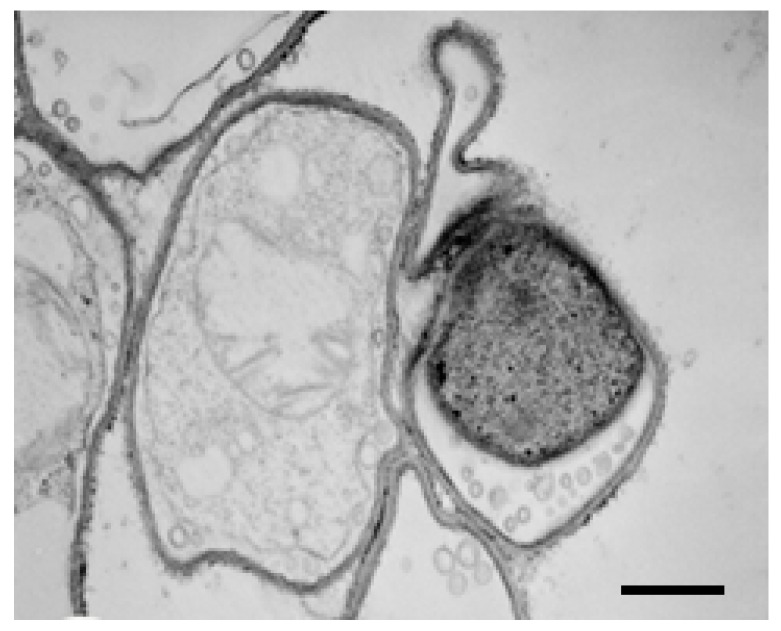
Cell death after treatment with 200 μg/mL phenylethylresorcinol; several cells are transparent and empty. TEM. Scale bar: 1 μm.

At the same time, the cytoplasm is very rich in ribosomes and smooth endoplasmic reticulum, which indicates an increase in protein and lipid metabolism. Some vacuoles have a relatively fine electron matrix, whereas others display a matrix with darker, irregularly shaped areas that are very opaque to electrons (Figure 6D). Finally, Figure 7 shows the presence of transparent and empty cells, a clear sign of cell death induced by the highest dose of the substance.

Another prominent observation is related to the cell wall, which is of great importance in fungus survival [11,16]. Cell wall structure is highly dynamic, changing constantly during cell division, growth and morphogenesis. It is generally accepted that the pattern of cell wall deposition is of primary importance in shaping the fungal cell and that interference or damage during the assembly of this structure negatively impacts the morphogenesis or life of the fungus [16]. Mares et al. [13] interpreted this increase in cell wall thickness as premature and abnormal aging of the fungus.

The cell wall has not been previously described as a target of resorcinols, whereas mitochondria, nucleic acids, proteins and, in particular, the endomembrane system are frequently reported as targets of these molecules [4,17,18].

## 3. Experimental Section

### 3.1. Microorganisms

The following seven fungal strains investigated in this study were purchased from the Centraal Bureau voor Schimmelcultures (CBS; Baarn, The Netherlands): *Arthroderma cajetani* Ajello, strain CBS 495.70; *Epidermophyton floccosum* (Hartz) Langeron and Milochevitch, strain CBS 358.93; *Trichophyton violaceum* Malmsten, strain CBS 459.61; *Trichophyton tonsurans* Malmsten, strain CBS 483.76; *Trichophyton mentagrophytes* (Robin) Blanchard, strain CBS 160.66; *Microsporum canis* Bodin, strain CBS 4727; and *Nannizzia gypsea* (Nann.) Weitzman, McGinnis, A.A. Padhye and Ajello, strain CBS 286.63. The remaining two strains were purchased from the Institute of Hygiene and Epidemiology-Mycology Laboratory (IHME; Brussels, Belgium): *Trichophyton rubrum* (Castellani) Sabouraud, strain IHME 4321 and *Microsporum gypseum* (Bodin) Guiart & Grigorakis, strain IHME 3999. All dermatophytes were maintained at 4 °C as agar slants on Sabouraud dextrose agar (SDA; Difco Laboratories, Inc., Detroit, MI, USA).

### 3.2. Chemicals

The tested substances—4-butylresorcinol (4-butyl-1,3-benzenediol, Acteosome), 4-hexyl-resorcinol (4-hexyl-1,3-dihydroxybenzene, Synovea) and racemic (±)phenylethylresorcinol (4-(1-phenylethyl)-1,3-benzenediol, Symwhite 377)—were purchased from Sigma-Aldrich SRL (Milano, Italy) and Symrise GmbH & Co. KG (Holzminden, Germany). Resazurin, used for fixation, and solvents were also purchased from Sigma-Aldrich SRL.

### 3.3. Growth Inhibition

Antifungal activity was determined as follows. Each test substance was dissolved in dimethyl sulfoxide (DMSO), and a suitable dilution was aseptically mixed with sterile SDA medium at 45 °C to obtain final concentrations of 5, 10, 20, 50, 100 and 200 µg/mL. The DMSO concentration in the final solution was adjusted to 0.1%. Controls were also prepared with equivalent concentrations (0.1% *v*/*v*) of DMSO. For the experiments, cultures were obtained by transplanting mycelium disks (10 mm in diameter) from a single mother culture in the stationary phase. They were incubated at 26 ± 1 °C on SDA on thin sheets of cellophane until the logarithmic growth phase. Subsequently, the cultures were transferred to Petri dishes with media containing 5, 10, 20, 50, 100 and 200 µg/mL of the three substances and incubated under growth conditions. The fungal growth was evaluated daily by measuring the colony diameters (in millimetres) for seven days beginning at the onset of treatment.

The percentage inhibition of growth was determined as the average of three different experiments. IC_50_ values were obtained for only the two substances that were active against the nine dermatophytes—phenylethylresorcinol and 4-hexylresorcinol—at concentrations of 5, 10, 20, 50, 100 and 200 µg/mL. The IC_50_ values were calculated as the average of three different experiments, and they indicate the concentration of the substance needed to inhibit the growth of the fungus by half.

### 3.4. Spore Germination Assay

#### 3.4.1. Evaluation of Spore Germination Inhibition

The efficacies of the three resorcinol derivatives tested were evaluated by resazurin assays using an optimized incubation time and spore density, as described by Romagnoli et al. [1]. A stock solution of each substance was prepared in DMSO. Then, phenylethylresorcinol, 4-hexylresorcinol and 4-butylresorcinol were added to test vials in duplicate at concentrations of 100 and 200 μg/mL. The test vials contained the substances to be tested, 10^5^ spore/mL (determined via previous evaluation), and 100 μL of resazurin stock solution in Sabouraud dextrose broth. The vials were covered, gently rotated horizontally to mix the contents and incubated in the dark at 24 °C for 120 h. Duplicate negative control vials containing 10 mL of medium and 100 μL of resazurin stock solution and duplicate positive control vials containing 10 mL of medium, 10^5^ spore/mL and 100 μL of resazurin stock solution were also processed. Fluorometric measurements and visual inspections were also conducted to evaluate spore germination. Fluorescence data were expressed as the percentage of resazurin reduced as a function of incubation time. After 24 h, the percentage of resazurin reduction was determined fluorometrically by measuring the fluorescence at 578 nm. The absorbance was read with a DU^®^ 530 Life Science UV/Vis spectrophotometer (Beckman Coulter^TM^, Brea, CA, USA) using a single-cell module.

The colours of the wells were also visually recorded (Figure 2). Blue was interpreted to indicate the absence of metabolic activity (no spore germination), whereas fluorescent pink was interpreted to indicate the presence of metabolic activity (spore germination). Purple was interpreted as a trailing result, reflecting the presence of some metabolic activity; however, prolonging the incubation time caused the purple colour to change to pink.

#### 3.4.2. TEM and SEM

The youngest hyphae of *M. gypseum* were chosen from untreated mycelia and from mycelia treated for 24 h with 20, 100 and 200 µg/mL phenylethylresorcinol for TEM and SEM analyses. Samples were fixed with 6% glutaraldehyde (GA) in 0.1 M sodium cacodylate buffer, pH 6.8, for 6 h at 4 °C. After rinsing in the same buffer solution, the samples for TEM were post-fixed for 15 h with 1% osmium tetroxide (OsO_4_) in the same buffer, dehydrated in a graded series of alcohol and embedded in Epon-Araldite resin. Sections were cut with an Ultratome III (LKB Instruments, Mount Waverley, Australia) stained with uranyl acetate and lead citrate and observed with an H-800 electron microscope at 100 kV (Hitachi, Altavilla Vicentina (VI), Italy),provided by the Electron Microscopy Center of Ferrara University). For SEM, samples were fixed with 6% GA in 0.1 M sodium cacodylate buffer, pH 6.8, for 6 h at 4 °C, briefly (1 h) post-fixed with 1% OsO_4_ in the same buffer, dehydrated in a graded series of alcohol, critical point-dried and gold-coated using an S 150 sputter coater (Edwards SpA, Cinisello Balsamo, Italy). SEM observations were collected using an EVO 40 instrument (Zeiss, Oberkochen, Germany) provided by the Electron Microscopy Center of Ferrara University).

## 4. Conclusions

In conclusion, select resorcinols already in use in the dermatology field for cosmetic applications were investigated and found to provide valuable activity against dermatophytic fungi. In particular, phenylethylresorcinol, widely used in the racemic form (under the trade name of Symwhite 377) in the dermo-cosmetic field as skin whitening, resulted of particular interest. These findings extend the application of resorcinols in medicine, especially for the treatment of human infections from soil or pets. Although the study of their mechanisms of action cannot be considered conclusive and requires further chemical investigations and evaluations using several different fungi, our results are of interest regarding the development of agents endowed with specific activities towards this class of emerging human pathogens. This study is based on a relatively safe approach that begins with the selection of molecules whose use is already established in the dermo-cosmetic field.

## Figures and Tables

**Table 1:** Percentage of growth inhibition induced by the three tested resorcinol derivatives against nine dermatophytes after seven days of treatment at 5, 10, 20, 50, 100 and 200 μg/mL. Each value is the mean of three measurements (all standard deviation values, not reported, were within 10%).

Compound	5 μg/mL	10 μg/mL	20 μg/mL	50 μg/mL	100 μg/mL	200 μg/mL
*M. gypseum*						
4-Hexylresorcinol	5.5	21.12	71.5	88.32	93.46	94.57
Phenylethylresorcinol	20.99	34.16	54.73	75.31	98.51	99.50
4-Butylresorcinol					25.00	33.33
*M. canis*						
4-Hexylresorcinol	17.5	32.5	82.5	85	92.68	97.5
Phenylethylresorcinol	+	+	10	47.5	94.74	95
4-Butylresorcinol					+	+
*T. violaceum*						
4-Hexylresorcinol	12.46	21.45	37.02	59.21	63.16	66.09
Phenylethylresorcinol	0.97	3.38	5.80	31.40	68.92	100
4-Butylresorcinol					+	+
*A. cajetani*						
4-Hexylresorcinol	18.11	43.31	69.29	74.02	78.13	82.29
Phenylethylresorcinol	+	13.77	39.86	67.39	84.56	100
4-Butylresorcinol					+	+
*T. mentagrophytes*						
4-Hexylresorcinol	31.48	43.52	81.08	82.41	87.04	88.89
Phenylethylresorcinol	5.88	10.92	36.13	93.28	99.16	100
4-Butylresorcinol					16.67	45
*E. floccosum*						
4-Hexylresorcinol	5.73	20.31	50.00	69.27	78.13	86.40
Phenylethylresorcinol	22.42	24.66	36.32	47.09	66.28	85.82
4-Butylresorcinol					12.61	19.82
*N. gypsea*						
4-Hexylresorcinol	13.61	19.73	53.74	76.19	83.67	85.47
Phenylethylresorcinol	11.56	16.76	31.21	50.87	82.66	97.78
4-Butylresorcinol					+	21.43
*T. rubrum*						
4-Hexylresorcinol	28.95	38.16	59.65	73.68	78.95	81.90
Phenylethylresorcinol	18.41	26.37	39.30	65.17	70.12	81.33
4-Butylresorcinol					+	5.86
*T. tonsurans*						
4-Hexylresorcinol	4.6875	18.75	75	85.94	92.19	93.10
Phenylethylresorcinol	12.5	14.0625	37.5	89.0625	100	100
4-Butylresorcinol					+	+

+ the substance showed an hormone-like effect with a fungal growth higher than that of the controls.

**Table 2:** Percent growth inhibition induced by phenylethylresorcinol on nine dermatophytes after seven days of treatment at 5, 10, 20, 50, 100 and 200 μg/mL. The percent growth inhibition induced by fluconazole is shown for comparison purposes. Each value is the mean of three measurements (all standard deviation values, which are not reported, were within 10%).

Compound	5 μg/mL	10 μg/mL	20 μg/mL	50 μg/mL	100 μg/mL	200 μg/mL
*M. gypseum*						
Fluconazole	12.34	34.54	55.12	76.01	80.23	85.40
Phenylethyl resorcinol	19.65	35.48	58.12	74.07	97.98	99.02
*M. canis*						
Fluconazole	17.12	26.48	39.95	61.87	80.43	88.51
Phenylethyl resorcinol	+	+	11.32	46.80	93.54	96.11
*T. violaceum*						
Fluconazole	36.76	67.65	83.78	81.33	97.08	94.65
Phenylethylresorcinol	1.13	5.43	6.02	27.58	69.05	100
*A. cajetani*						
Fluconazole	0.0	0.0	0.0	0.0	0.0	8.67
Phenylethylresorcinol	+	13.77	39.86	67.39	84.56	100
*T. mentagrophytes*						
Fluconazole	62.46	79.67	100	100	100	100
Phenylethylresorcinol	7.31	11.96	35.56	94.00	98.00	100
*E. floccosum*						
Fluconazole	98.00	100	100	100	100	100
Phenylethylresorcinol	21.79	25.11	39.87	46.87	68.53	87.67
*N. gypsea*						
Fluconazole	0.0	0.0	0.0	0.0	1.09	5.78
Phenylethylresorcinol	13.43	18.99	35.11	50.95	83.74	98.89
*T. rubrum*						
Fluconazole	13.13	25.66	40.44	58.76	68.68	76.06
Phenylethylresorcinol	17.15	28.55	39.92	64.69	72.34	84.48
*T. tonsurans*						
Fluconazole	27.77	48.56	44.12	72.33	71.33	79.90
Phenylethylresorcinol	13.17	16.44	35.57	89.74	100	100

+ the substance showed an hormone-like effect with a fungal growth higher than that of the controls.

**Table 3:** IC_50_ values of 4-hexylresorcinol and phenylethylresorcinol against nine dermatophytes. Each value is the mean of three measurements (all standard deviation values, which are not reported, were within 10%).

Dermatophyte	4-Hexylresorcinol	Phenylethylresorcinol
	IC_50_ μg/mL	
*M. gypseum*	18.77	11.42
*M. canis*	14.02	43.47
*T. violaceum*	48.39	54.60
*A. cajetani*	16.22	30.87
*T. mentagrophytes*	12.97	24.68
*E. floccosum*	29.42	37.40
*N. gypsea*	24.72	33.64
*T. rubrum*	16.22	31.42
*T. tonsurans*	20.09	23.24

**Table 4:** Resazurin assay. Percentage inhibition of spore germination after treatment with three resorcinols at doses of 100 and 200 μg/mL.

Compound	Concentration	% Inhibition of Spore Germination
4-Hexylresorcinol	100 μg/mL	100
	200 μg/mL	100
Phenylethylresorcinol	100 μg/mL	100
	200 μg/mL	100
4-Butylresorcinol	100 μg/mL	13.5 ± 0.8
	200 μg/mL	80.2 ± 1.1
